# Perspectives on Involuntary Treatment of Anorexia Nervosa

**DOI:** 10.3389/fpsyt.2020.533288

**Published:** 2020-10-21

**Authors:** Loa Clausen

**Affiliations:** ^1^Department of Child and Adolescent Psychiatry, Aarhus University Hospital Psychiatry, Aarhus, Denmark; ^2^Department of Clinical Medicine, Aarhus University, Aarhus, Denmark

**Keywords:** involuntary treatment, anorexia nervosa, severe and enduring anorexia nervosa, coercion, eating disorders, restraint

## Abstract

Involuntary treatment of anorexia nervosa is an option in cases in which the patient's life or other people's lives are at risk or, in some countries, to prevent the deterioration of the illness. Involuntary treatment is often regarded as controversial and has been intensely debated, although typically with few references to documented knowledge. This paper provides a research perspective of the topic by examine data in the field of the involuntary treatment of anorexia nervosa to pinpoint present knowledge as well as areas demanding clinical action or research attention. The prevalence of involuntary treatment in general as well as specific measures is described and possible early markers of patients at risk of involuntary treatment are discussed. Studies including patients' perspectives of involuntary treatment show the complexity of this treatment, its initiation, and its consequences. To qualify future discussions, improve current practice, and minimize involuntary treatment in general as well as on an individual level, at least four areas need attention: (i) the present specific symptoms of anorexia nervosa and their imminent consequences, (ii) illness history, (iii) overall psychiatric symptoms and general functioning, and (iv) contextual sphere of the patient. In particular, the last two require attention from both clinicians and researchers. Furthermore, critical evaluation of the attitudes of both patients and health care professionals toward each other and the treatment is recommended.

## Introduction

Anorexia nervosa is an illness with an increased mortality rate from both natural and unnatural causes of death (1, 2). The characteristics of anorexia nervosa are self-induced low weight, a disturbed body image, and a fear of weight gain (3). Patients with severe and enduring anorexia nervosa are additionally characterized by being ill for a long time and having significant eating disorder symptoms as well as being resistant or ambivalent toward treatment (4). Hence, these patients are specifically at risk of being treated against their will based on both the dangerous and the deterioration criteria (5).

As described below, involuntary treatment is usually evaluated negatively by patients, professionals, and relatives (6–10). Inpatient care must thus always aim to find alternative strategies and interventions to involuntary treatment, reducing it whenever possible without neglecting its lifesaving purpose and outcome. To do this, up-to-date knowledge on the involuntary treatment of anorexia nervosa is needed.

### Prevalence and Predictors

The involuntary treatment of anorexia nervosa, which occurs in 13 to 44% of admissions, is associated with severe psychiatric symptoms, comorbidity, previous admissions, and long illness duration (11, 12).

The significance of preadmissions and illness duration on involuntary treatment are well-established and intuitive as they represent different aspects of illness severity. By contrast, comorbidity and severe psychiatric symptoms are more complex and imprecise terms that are not immediately applicable to clinical practice.

A recent register-based study found that the comorbidity associated with the involuntary treatment is caused by all the main diagnostic groups except intellectual disability. Behavioral and emotional disorders with onset in childhood show a weak association, whereas schizophrenia spectrum disorders, personality disorders, and autism spectrum disorders are the strongest predictors along with age at onset and earlier admissions (13). However, while the association between schizophrenia spectrum disorder and personality disorders and involuntary treatment is well-described within general psychiatry (14–16), the link between autism spectrum disorders and involuntary treatment among psychiatric patients is less clearly examined. However, people with developmental disorders including autism spectrum disorders have been found to have a similar increased risk of involuntary treatment as patients with schizophrenia (16).

Comorbid psychiatric illness is well-described in patients with anorexia nervosa (17) and has been suggested as a defining criterion in severe and enduring anorexia nervosa (18). However, the role of comorbid illness remains unknown. For example, it is unclear whether it raises the risk of involuntary treatment, because (i) comorbidity increases the complexity of overall mental functioning, (ii) the comorbid illness symptoms themselves prompt the involuntary episode, or (iii) the complexity of psychiatric symptoms complicates clinical decisions, thereby increasing the risk of an inexpedient therapeutic response.

### Different Involuntary Measures

Involuntary admissions, detentions, and nasogastric tube feeding have been described in relation to anorexia nervosa (19–21). However, one register-based study finds that all involuntary measures are used with patients with anorexia nervosa including medication and mechanical or physical restraint (13). Because the use of these more intrusive involuntary measures not directly relates to the symptoms of anorexia nervosa has been described in relation to compulsory tube feeding in two single case studies of anorexia nervosa (22, 23), we do not expect the results to reflect a country-specific practice. However, it remains relevant to examine whether the use of such measures is common across countries, as legislation on the use of involuntary treatment varies globally and cultural aspects have been shown to affect the frequency and type of involuntary measures (5, 24–27). In addition, questions on the extent to which patient-specific, illness–specific, and contextual factors affect specific involuntary measures need to be answered as well as the impact of these measures on patients.

### Attitude Toward Treatment

Attitude toward treatment is an important topic when discussing the involuntary treatment of anorexia nervosa, as these patients often lack the motivation to change or refuse to accept they have a treatment need (28). Their decision-making capacity and their attitudes toward treatment are affected by the ego syntonic nature of the disease (23, 29, 30). At the intrapsychic level, Seed et al. (23) argue that the self is occupied by the illness and Tan et al. (31) describe how patients' value system changes because of anorexia nervosa, resulting in weight-related issues overshadowing other aspects of their life.

Motivation to change and the perception of treatment need have both been found to improve during treatment. Guarda et al. (32) found that 41% of those rejecting an admission need at the time of admission changed their stance after 2 weeks of inpatient treatment and acknowledged a treatment need. Motivation to change has also been shown to increase gradually during admission (33). These changes could reflect an improvement in the decision-making capacity found in a third of patients admitted with anorexia nervosa (29) or patients giving up their resistance to treatment (23). Hence, changes in motivation and the perception of admission need have been a crucial argument for persuading patients into admission. However, the majority of patients with anorexia nervosa, although accepting they are not formerly coerced, state that they do experience a high degree of perceived pressure, informal coercion, and procedural justice (32–34). This has been reported by patients with increased eating disorder psychopathology (34), younger patients, and patients with mild weight loss (33).

Although the use of such informal coercion interventions seems less dramatic or intrusive than formal coercion, it does make the patient feel a loss of autonomy, which is why voluntary and collaborative admission is ideal through therapeutic alliances, transparent dialogue, and motivational interventions whenever possible (35, 36). Furthermore, Seed et al. (23) argue that professionals should take the position of “safe-uncertainty” (37), where several explanations and solutions to a given problem may exist simultaneously, where the therapist is less authoritarian and less of an expert, and where the patient is given a major role in the decision-making process. While this is difficult to uphold in the acute situation where involuntary treatment is deemed necessary and initiated, it does seem important before and after involuntary episodes to prevent future episodes or decrease the negative impact of involuntary treatment on patients, relatives, and professionals. In this way, in addition to the attitude of the patient, the attitude of health-care professionals toward the patient and his/her treatment is crucial if we are to understand and decrease involuntary measures in the future.

### Patients' Perception of Involuntary Treatment

Patients' perception of the precursors to or reasons for involuntary episodes augment clinical research that mainly focuses on patient characteristics and typically overlooks the importance of the attitude and action of health-care professionals, including their use of control, and patients' need to protest (about the treatment or environmental circumstances) (26, 38, 39).

Furthermore, although patients with anorexia nervosa report involuntary treatment interventions as necessary, life-saving, and a sign of caring relations, they mainly see them as actions of punishment and something that should either be short-lasting or even prohibited (23, 40, 41). Some patients argue for the use of involuntary treatment earlier in the illness course, whereas other argue for the right to choose to die from anorexia nervosa (40). Reports of this typically negative perception of involuntary treatment are well-known from general psychiatric patients also (38, 39, 42). The impact of different involuntary measures on these patients seems to vary by measures, with seclusion and restraint having an especially negative impact (6, 39, 42). The subjective implications of nasogastric feeding specifically have in a small qualitative study been reported to increase rebellious behavior as well as involuntary measures such as restraint and forced medication (23).

Hence, studies including patients' perception of involuntary treatment provide information on the relational and contextual factors influencing the risk of involuntary treatment. Such studies are thus warranted to understand the dynamics initiating and escalating involuntary treatment episodes. In addition, clinicians continuously need to be aware of these dynamics if they are to decrease the use of involuntary treatment.

## Discussion of Future Directions in Research and Treatment

To understand the overall use of involuntary treatment, decrease it, qualify discussions, and improve practice, we must focus on at least four areas. The first area is the present specific anorexia nervosa symptoms of patients, including (i) the somatic status and present physical symptoms of anorexia nervosa, as well as imminent consequences, at least in countries in which involuntary treatment might be initiated to prevent a deterioration of the illness (5) and (ii) the psychopathological aspect of anorexia nervosa, including the value system of patients, insight into their situation, decision-making capacity, and degree to which anorexia nervosa occupy the self.

Second, illness history includes important markers of the risk of involuntary treatment, with a longer duration of illness, older age at first diagnosis, and history of earlier treatment as important predictive factors (11). The association between involuntary treatment and longer duration or number of admissions can be explained as the influence of illness severity as well as the patient's earlier experience and attitude toward treatment. However, the effect of the attitude of health-care professionals must not be neglected as their knowledge of the patient as having a resistant illness may increase their expectations of an involuntary treatment need (10).

The third area to consider is the patient's general functioning and psychiatric symptoms, including self-harm, sexual/physical abuse, and other mental illnesses, especially autism spectrum disorders, schizophrenia and personality disorders (11, 13). These disorders all include some level of basic disturbed and inflexible cognitive and social functioning (43–46) and their coexistence in patients with anorexia nervosa is expected to affect treatment and the relationship with health-care professionals, consequently also impacting on the treatment outcome, including the risk of involuntary treatment. Thus, a thorough assessment of the central comorbid disorders and basic cognitive, communicative, and relational abilities of patients is important in severe anorexia nervosa. Similarly as coexisting psychopathologies affect the relational sphere the match between patient and treatment or therapist might need to be examined, which leads us to the fourth area.

Finally, the contextual area including the exploration of the influence of legislation, systems, relations, and treatment has been found to be associated with involuntary treatment (5, 10, 14). Involuntary episodes might be the manifestation of power over the individual/illness/situation, of powerlessness, or the anxiety of health-care professionals or the patient (23, 38, 39, 47). The expectations of the patient or health-care professionals affect the risk of involuntary admission (10, 48). Therefore, analyzing the build-up to an episode of involuntary measures is an important clinical task to understand and prevent involuntary episodes. Besides intra-clinical factors, examining the influence of the patient's social support and network, which has not thus far been studied in patients with anorexia nervosa, has been found to be an important risk factor of involuntary admissions in acute psychiatry (49).

Lastly, attention must be directed toward the outcome of involuntary treatment. Traditional positive outcome markers such as remission and symptom reduction are insufficient, as involuntary treatment depends upon dangerousness or deterioration criteria, which is why decreased mortality and stable physiology and symptoms might be more relevant markers of outcomes. Unfortunately, research on the effect of involuntary treatment in anorexia nervosa is in its infancy. The findings on the mortality rate are mixed and not applicable as studies compare rates of patients treated involuntary with those treated voluntarily (19, 50) even though involuntary measures can be initiated only when deemed needed in contrast to voluntary treatment.

## New Treatments and the Ethical and Legal Complex of Involuntary Treatment

The exploration of these four areas is complicated by important ethical and legal issues. It is possible to fail the Hippocratic Oath (first, do no harm) both by initiating involuntary treatment and by not initiating it (51, 52). Commitment laws are justified by the caretaking of the patient or society and overrule normal rights to consent to or refuse treatment (5). Substituting the patient's personal right to decide on his/her own life and treatment is controversial, however, the alternative is the loss of life or loss of the right to die. Decisions on use or non-use of involuntary treatment are extremely complex, hence, studies of legal and ethical issues are important (36, 52–55).

For patients with short-term illnesses, we have to do what we can, even if that includes involuntary treatment in the most severe cases, knowing that anorexia nervosa affects their illness perception and that (early) weight gain is a predictor of improved cognition as well as symptom outcome (56–58). For patients that have been challenged by anorexia nervosa in the long term, with unsuccessful treatment and long-lasting suffering, treatment choice is more complex (59). Studies including established treatments of anorexia nervosa, i.e., Cognitive Behavioral Therapy for Eating Disorders, Maudsley Model of Anorexia Nervosa Treatment for Adults, Specialist Supportive Clinical Management, or modifications of these, have found that symptom outcome improves in some patients with long-term anorexia nervosa (59–61). However, in general new treatments of severe and enduring anorexia nervosa include suggestions to minimize or even dismiss the focus on eating disorder symptoms and instead focus on quality of life (18, 60, 62–64). Palliative care could be considered and admission should in some cases only be initiated with consent and for symptom interruption rather than to normalize weight (51, 65). However, studies evaluating such treatment approaches are still scarce (60, 66) although needed if we are to improve treatment for the most severe patients with increased risk of involuntary treatment.

## Conclusion

The involuntary treatment of anorexia nervosa is a complex area and further research including quantitative and qualitative studies is needed. Studies focusing on outcomes, patient-specific and contextual factors, and precipitating and processual factors are needed to reduce involuntary treatment, by, for example, the early identification of patients at risk of involuntary treatment and by identification of episodes escalating to include involuntary measures.

Patient characteristics such as severe eating disorder symptoms, psychiatric comorbidity, and illness history are important as involuntary treatment might be more justified in cases with shorter durations and less in cases with long illness duration and years of unsuccessful treatment (23). Understanding the underlying individual psychopathology can thus be vital, including the possible cognitive, communicative, and relational difficulties.

The contextual factors relevant for involuntary treatment are many and often not well-described. A critical examination of how we as therapists contribute or how our clinical culture contributes to the initiation or escalation of involuntary treatment is important. This might lead to new perspectives on episodes of involuntary treatment. Kendall (36) suggests more dialogue with more autonomy and power passed to the patient in the decision-making process, Seed et al. (23) suggest a longer-term recovery approach with a position of more “safe-uncertainty,” and several studies suggest focus on quality of life instead of eating disorder symptoms (51, 62, 64). Traditional eating disorder treatment usually focuses on normalizing eating and weight, often with use of non-negotiables (67). However, this might not be the right approach in cases with severe and enduring anorexia nervosa, because this approach might result in disrespecting the patient's wishes and autonomy or exacerbating rigidity and protest behavior, especially in cases with a history of several unsuccessful treatment attempts. Professionals' compassionate care (68) and containment of patients' negative emotions (69) are basic treatment elements that need to be stressed in eating disorder treatment along-side the well-established focus on symptom reduction (57). In cases with several failed treatment attempts, adjustment must be done and clinicians are obliged to search for new approaches, including the right dose of patience, containing and compassion, along with goals for weight gain or stabilization, meal support, guidance and dialogue in the treatment. Finally, individualized approaches tailored to a person's specific characteristics, psychological capacity, treatment history, and social support are important, as the consideration of involuntary treatment guarantees a complex case.

## Author Contributions

The author confirms being the sole contributor of this work and has approved it for publication.

## Conflict of Interest

The author declares that the research was conducted in the absence of any commercial or financial relationships that could be construed as a potential conflict of interest.
